# Prosthetic valve endocarditis presenting with back pain alone: A case report

**DOI:** 10.1002/jgf2.316

**Published:** 2020-05-14

**Authors:** Toshihiro Sato, Shinichiro Morioka, Takato Nakamoto, Keiji Nakamura, Yusuke Irisawa, Tetsuya Horai, Norio Ohmagari

**Affiliations:** ^1^ Disease Control and Prevention Center National Center for Global Health and Medicine Tokyo Japan; ^2^ Department of Cardiovascular Surgery National Center for Global Health and Medicine Tokyo Japan

**Keywords:** blood culture, case report, physical examination, polymyalgia rheumatica, prosthetic valve endocarditis

## Abstract

We report a case of a 64‐year‐old woman with a past medical history (PMH) of prosthetic valve replacement 7 months prior to admission, who presented with only right back pain. Physical examination revealed one conjunctival spot hemorrhage and a mild systolic murmur. Blood cultures were positive for methicillin‐resistant *Staphylococcus epidermidis*. Trans‐esophageal echocardiography revealed aortic valve vegetations; hence, a diagnosis of prosthetic valve endocarditis (PVE) was made. Clinical presentation of infective endocarditis varies and sometimes mimics that of polymyalgia rheumatica. The patient was diagnosed as PVE considering the whole clinical picture including the patient's PMH, physical examination, and blood cultures.

## Graphical abstract

This case report demonstrates that some cases of infective endocarditis (IE) can show musculoskeletal symptoms alone, and it is difficult to distinguish it from polymyalgia rheumatica and other diseases.

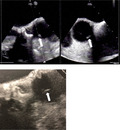

## 1 INTRODUCTION

Infective endocarditis (IE) is mentioned as an important candidate in the differential diagnosis of a variety of symptoms. Fever is reported to be seen in 90% of cases.^1^ IE is also known to cause musculoskeletal symptoms, and the frequency is about 44%.^2^ Clinical presentation of polymyalgia rheumatica (PMR) sometimes mimics that of IE because PMR causes musculoskeletal pain, typically in middle‐aged and elderly women.^3^ We report a case of a middle‐aged woman with prosthetic valve endocarditis whose complaint was back pain alone without fever.

## 2 CASE REPORT

A 64‐year‐old woman experienced right back pain, which appeared 6 days prior to admission (PTA); she visited the emergency outpatient unit of our hospital 5 days PTA. Her right back pain was around the right costovertebral angle, and the pain did not migrate. She had no fever or any other symptoms. She had an aortic valve replacement for severe aortic stenosis 7 months PTA. She did not have any postsurgical complications, and cefazolin was used for 3 days during the perioperative period. Her body temperature was 36.9°C; blood pressure was 114/74 mm Hg on the right arm and 114/81 mm Hg on the left arm. On physical examination, conjunctival hemorrhage or cardiac murmur was not appreciated. A laboratory test revealed that white blood cell (WBC) count was elevated to 8,830/µL, and C‐reactive protein (CRP) level was elevated to 10.12 mg/dL (Table 1).

**TABLE 1:** Laboratory results of the patient. (A, Laboratory data 5 days prior to admission. B, Results of the urinary analysis on the 1st day of admission)

(A) Hematology		Biochemistry			
WBC	8830/μL	Alb	3.6 g/dL	ESR	50 mm/h
Neutrophils	79.2％	BUN	10.9 mg/dL	ferritin	137 ng/mL
Lymphocytes	14.3％	Cr	0.63 mg/dL	ANA	negative
		T‐Bil	0.6 mg/dL	c‐ANCA	<0.5 IU/mL
RBC	446 × 10^6^/μL	AST	20 U/L	p‐ANCA	0.5 IU/mL
Hb	12.8 g/dL	ALT	14 U/L	MMP‐3	51.3 ng/mL
Ht	38.4％	LDH	258 U/L	anti‐CCP	0.6 U/mL
Plt	22.7 × 10^3^/μL	ALP	374 U/L		
		CK	45 U/L		
		Na	142 mEq/L		
		K	4.6 mEq/L		
		Cl	103 mEq/L		
		CRP	10.12 mg/dL		

(B)				
pH	6.5 sediment	RBC	1‐4	HPF
Protein	Negative	WBC	<1	HPF
Glucose	Negative	Bacterial	‐	
Blood	+/−	Hyaline	2+	
Leukemia	Negative			

Abbreviations: Alb, albumin; ALP, alkaline phosphatase; ALT, alanine aminotransferase; ANA, antinuclear antibody; ANCA, antineutrophil cytoplasmic antibody; anti‐CCP, anti‐cyclic citrullinated peptide antibody; AST, aspartate aminotransferase; BUN, blood urea nitrogen; CK, creatine kinase; Cr, creatinine; CRP, C‐reactive protein; ESR, erythrocyte sedimentation rate; LDH, lactate dehydrogenase; MMP‐3, matrix metalloproteinase‐3; Plt: platelets; RBC, red blood cell; T.Bil, total bilirubin; WBC, white blood cell.

In the emergency unit, we were concerned about aortic dissection, infected arterial aneurysm, and purulent arthritis; hence, enhanced CT of the chest and abdomen was performed; however, there were no abnormal findings. Considering PMH and physical examination, we performed blood cultures. Four days PTA, methicillin‐resistant *Staphylococcus epidermidis* (MRSE) was detected from two sets of blood cultures. We recommended hospitalization, but she refused and insisted on going home. We repeated blood cultures and let her return home. No antibiotics were started. MRSE was positive in two sets of the repeated blood cultures; hence, she was admitted to our hospital.

On admission, her body temperature was 36.1°C. On physical examination, we confirmed one punctate spot hemorrhage in the palpebral conjunctiva and a systolic murmur (grade 2/6) at the left sternal border. Costovertebral angle tenderness, spinal tap pain, and rash on her trunk, fingers, or toes were not appreciated. A urinalysis revealed that there were no bacteriuria, pyuria, and hematuria.

**FIGURE 1:**
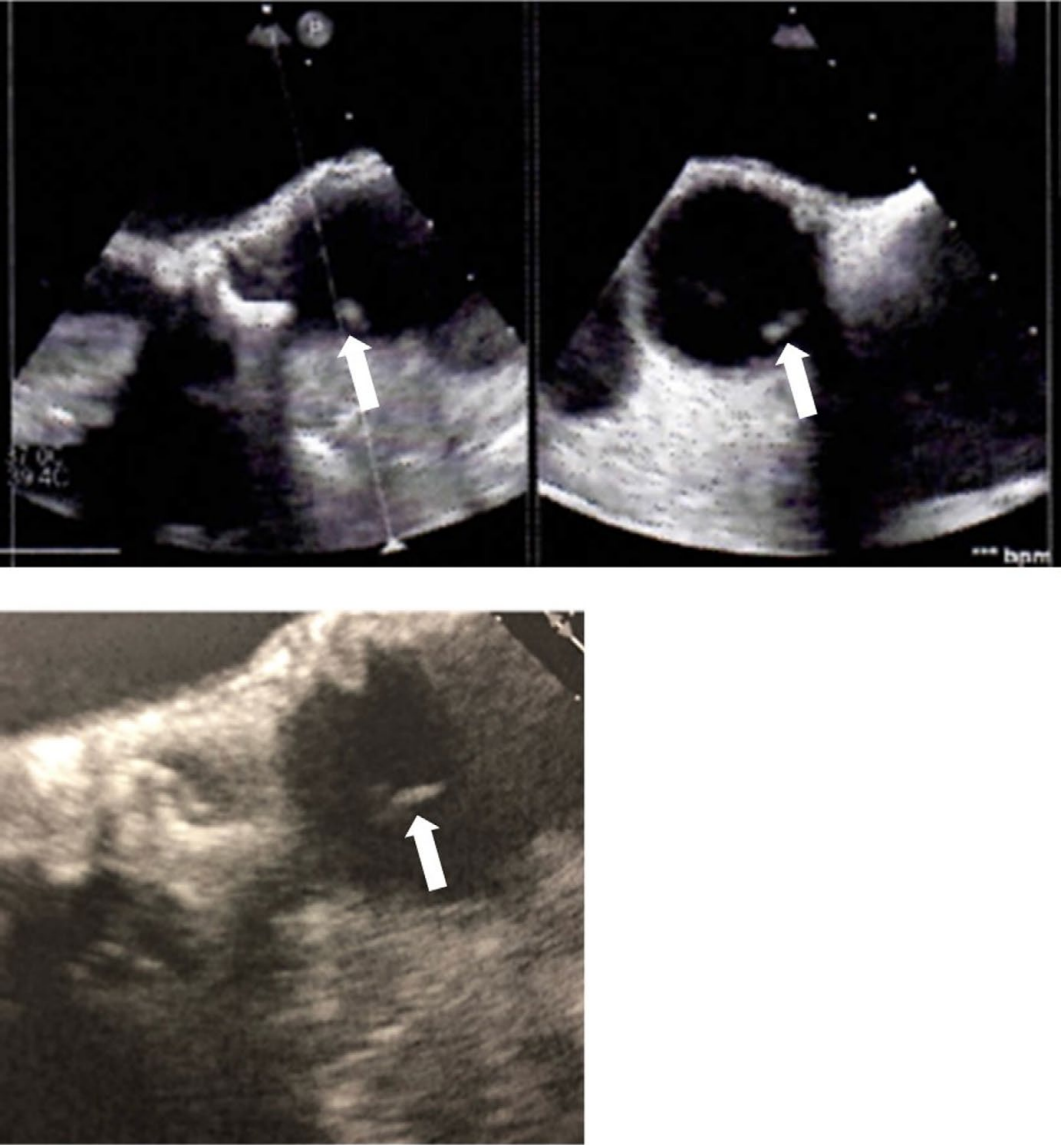
Trans‐esophageal echocardiogram showing a vegetation attached to the aortic valve (white arrows, left: mid‐esophageal long‐axis view; right: short‐axis view; under: mid‐esophageal long‐axis view magnified image)

Although PMR was an important differential diagnosis from the chief complaint of right back pain in the patient, prosthetic valve endocarditis (PVE) was more likely considering the PMH of aortic valve replacement, physical examination findings such as systolic cardiac murmur, conjunctival hemorrhage, and, especially, blood culture results positive for MRSE. Trans‐thoracic echocardiography demonstrated no vegetations; however, trans‐esophageal echocardiography (TEE) revealed vegetations (2.1 × 5.3 mm) on the aortic valve, though paraleakage, valve destruction, or perivalvular abscess was not detected (Figure 1). These findings met the two major points of Duke's criteria. A diagnosis of PVE was made.

Vancomycin was started immediately on day 1. Aminoglycoside was resistance to MRSE in sensitivity testing. Though the repeated blood cultures on day 5 were positive, they turned negative on day 8. We considered that the bacterial burden decreased, and rifampicin was added on day 13. On day 19, vancomycin was switched to teicoplanin because of leukopenia, thrombocytopenia, and fever, and rifampicin was discontinued because of the subsequent rash. On day 20, we performed blood cultures, and they were negative. We considered that aortic valve re‐replacement was relatively indicated because the size of the vegetation did not change in the repeated TEE on day 19 even though the size was not >10 mm. However, we needed to postpone the surgical procedure due to leukopenia and thrombocytopenia possibly caused by vancomycin infusion. We performed aortic valve re‐replacement on day 33. Because the postoperative clinical course was good, the patient was discharged on day 50.

## 3 DISCUSSION

This case demonstrates the following: (a) Some cases of IE can show musculoskeletal symptoms alone, which makes it difficult to diagnose; (b) comprehension of the whole clinical picture including the PMH of the patient, careful physical examination, and collection of blood samples for culture helped make a definitive diagnosis and start treatment without delay.

It has been known that patients with IE develop various symptoms and that they sometimes present with musculoskeletal manifestations such as muscular pain and arthralgia. It is necessary to distinguish IE from orthopedic diseases and collagen vascular diseases including PMR. Churchill et al reported that the incidence of musculoskeletal manifestations was around 44%, and the percentage of cases in which the initial symptom was musculoskeletal manifestations alone was reported to be around 15%.^2^ We considered that patients with IE sometimes present with back pain, and it held true in our case.

Polymyalgia rheumatica is known as a typical disease‐causing back pain in middle‐aged and elderly women and is often difficult to distinguish from IE. Several cases of IE with symptoms similar to PMR have been reported.^4^, ^5^, ^6^ It is extremely important to exclude IE when we make a diagnosis of PMR.

Considering the differential diagnoses from the PMH, careful physical examination and blood cultures helped us to make a diagnosis of PVE in this case. This patient had undergone aortic valve replacement seven months PTA. The risk of IE after valve replacement is reported as 1 to 3% within 1 year of surgery. It is particularly high within 6 months of surgery but diminishes over time.^7^ This case was considered as being in a relatively high‐risk group. We confirmed one punctate spot hemorrhage in the palpebral conjunctiva, a mild systolic murmur. Considering the high sensitivity of cardiac murmurs of 80%–85% reported in IE,^8^ the cardiac murmur was an important finding. However, we were unaware whether the murmur was preexisting due to a lack of medical records. In addition to it, we could not identify the entry of the microorganism.

Although fever is a common symptom seen in 90% of patients with IE,^1^ this patient had no fever. Blood cultures played an important role in the diagnosis of this case. The sensitivity of blood cultures for IE is about 90%.^9^ We suggest that blood cultures be taken during examination for a patient who has had valvular surgery and who presents with musculoskeletal symptoms and elevated inflammatory markers.

It is very important to make an accurate diagnosis of IE without any delay, because delay in diagnosis may lead to severe outcomes or complications. The mortality rate of IE is reported to be approximately 20% within the first 30 days.^10^ Auzary et al reported a case of IE, which was initially misdiagnosed as giant cell arteritis and used prednisone as treatment.^4^ In this case, we primarily suspected IE by considering the PMH, and careful physical examination in addition to eliciting the history of the back pain. As a result, we were able to start antibiotics without any delay.

## 4 CONCLUSION

In this case, the patient presented with musculoskeletal manifestations alone, which made it difficult to make a diagnosis. However, we primarily suspected IE by considering the patient's recent history of valve replacement surgery, careful physical examination, and blood cultures. As a result, we were able to start antibiotics without any delay. Comprehension of the global clinical picture including PMH, physical examination, and performing blood cultures was critical for the diagnosis.

## CONFLICT OF INTEREST

The authors have stated explicitly that there are no conflicts of interest in connection with this article.

## References

[1] Durante‐Mangoni E , Bradley S , Selton‐Suty C , Selton‐Suty C , Tripod MF , Bouza B , et al. Current features of infective endocarditis in elderly patients: results of the international collaboration on endocarditis prospective cohort study. Arch Intern Med. 2008;168:2095–103. 18955638 10.1001/archinte.168.19.2095

[2] Churchill MA , Geraci JE , Hunder GG . Musculoskeletal manifestations of bacterial endocarditis. Ann Intern Med. 1977;87:754–9. 145198 10.7326/0003-4819-87-6-754

[3] Salvarani C , Cantini F , Boiardi L , Hunder GG . Polymyalgia rheumatica and giant‐cell arteritis. N Engl J Med. 2002;347(4):261–71. 12140303 10.1056/NEJMra011913

[4] Auzary C , Le Thi HD , Delarbre X , Sbai A , Lhote F , Papo T , et al Subacute bacterial endocarditis presenting as polymyalgia rheumatica or giant cell arteritis. Clin Exp Rheumatol. 2006;24:S38–40. 16859595

[5] González‐Gay MA , García‐Porrúa C , Salvarani C , Olivieri I , Hunder GG . Polymyalgia manifestations in different conditions mimicking polymyalgia rheumatica. Clin Exp Rheumatol. 2000;18:755–9. 11138344

[6] De Socio GV , Mencacci A , Bini P , Pasticci MB . Fusobacterium nucleatum endocarditis mimicking polymyalgia rheumatica. South Med J. 2009;102:1082–4. 19738533 10.1097/SMJ.0b013e3181b4e5b8

[7] Calderwood SB , Swinski LA , Waternaux CM , Karchmer AW , Buckley MJ . Risk factors for the development of prosthetic valve endocarditis. Circulation. 1985;72:31–7. 4006134 10.1161/01.cir.72.1.31

[8] Kiefer TL , Bashore TM . Infective endocarditis: a comprehensive overview [internet]. Rev Cardiovasc Med. 2012;13:e105–20. 23160159 10.3909/ricm0633

[9] Washington JA . The microbiological diagnosis of infective endocarditis. J Antimicrob Chemother. 1987;20:29–39. 3316162 10.1093/jac/20.suppl_a.29

[10] Vincent LL , Otto C . Infective endocarditis: update on epidemiology, outcomes, and management. Curr Cardiol Rep. 2018;20:86. 30117004 10.1007/s11886-018-1043-2

